# A novel type of *Plasmodium* sporozoite transcellular migration

**DOI:** 10.1186/1475-2875-9-S2-P41

**Published:** 2010-10-20

**Authors:** Kathleen E Rankin, Joana Tavares, Nicole S Struck, Robert Menard, Volker T Heussler, Rogerio Amino

**Affiliations:** 1 Molecular Parasitology, Bernhard Nocht Institute for Tropical Medicine, Hamburg, Germany; 2 Unité de Biologie et Génétique du Paludisme, Institut Pasteur, Paris, France

## 

During liver-stage malaria infection, *Plasmodium* sporozoites must invade a hepatocyte in order to produce thousands of merozoites, the stage that infects erythrocytes and causes malarial pathology. One of the first steps of hepatocyte infection is the invagination of the host cell membrane, which shelters the parasite within a parasitophorous vacuole (PV) and allows parasite development inside the host cell. Sporozoites also have another peculiar type of cell invasion, in which the parasite breaches the host cell plasma membrane and transmigrates, free in the cytoplasm, through the host cell. This cell wounding and crossing process is termed cell traversal. Here, using live-cell imaging techniques, fluorescent markers of the host cell plasma membrane and a mutant parasite lacking cell traversal activity, we describe a new type of sporozoite transcellular migration. We have observed wild-type *Plasmodium berhgei* sporozoites moving through hepatocytes while surrounded by host cell membrane. Additionally, a small percentage of sporozoites drag host cell membrane with them when they leave hepatocytes, forming aggregates of membrane we have termed membrane "bobbins." Membrane-surrounded migrating sporozoites and membrane bobbins are also observed during transmigration of primary liver sinusoidal endothelial cells. As cell traversal deficient sporozoites are still able to move across the cell surrounded by host cell plasma membrane, we define this new transcellular migration as being distinct from cell traversal.

We hypothesize that this form of transmigration starts with the PV membrane formation similar to invasion with the exception that these sporozoites continue migration, ultimately leaving the cell by membrane fusion or disruption of the vacuole membrane and the plasma membrane.

